# Food consumption behaviour and self-perceived nutrition knowledge: a case study of students with limited formal nutrition education in pre-university schooling

**DOI:** 10.1017/S1368980026102468

**Published:** 2026-04-10

**Authors:** Wing-Fu Lai, Haotian Zhang, Sreekanth Reddy Obireddy

**Affiliations:** 1 School of Food Science and Nutrition, University of Leeds, UK; 2 School of Life and Health Sciences, The Chinese University of Hong Kong (Shenzhen), China; 3 Department of Chemistry, Sri Krishnadevaraya University, India

**Keywords:** Students, Food choice behaviour, Nutrition knowledge, Illusion of knowing, Education

## Abstract

**Objective:**

To investigate food consumption behaviour and self-perceived nutrition knowledge among university students, and to draw implications for nutrition education in contexts where formal nutrition education before university is limited.

**Design:**

A mixed-methods approach was adopted. A survey was first conducted to examine participants’ food consumption behaviour and self-perceived nutrition knowledge. Thirty-four participants were then invited to take part in semi-structured interviews to gain more in-depth insights into their self-declared knowledge and related behaviours.

**Setting:**

Universities in China, representing a context of limited formal nutrition education in pre-university schooling.

**Participants:**

190 university students.

**Analysis:**

Interview transcripts were reviewed to verify participants’ self-declared nutrition knowledge and identify misconceptions or gaps in understanding. Questionnaire data were analysed using descriptive statistics.

**Results:**

Students with higher education levels reported paying more attention to nutrition labels and selecting healthier snacks. However, interviews revealed that students who claimed to read nutritional claims during food purchases often misunderstood the meaning of sugar and fat content information. A significant ‘illusion of knowing’ was observed, and participants generally lacked awareness of authoritative food standards.

**Conclusion and implications:**

Illusion of knowing is common among students who have not received formal systematic nutrition education. Nutrition education programmes should prioritise raising students’ understanding of basic food concepts and improving their ability to interpret nutrition information accurately, as part of broader health promotion efforts.

With continuous social development worldwide, the nutritional needs and awareness of the general public are increasing. Nutrition literacy influences food choices, and unhealthy dietary patterns are associated with obesity, elevated cardiovascular risks and impaired cognitive performance^(1)^. Evidence suggests that nutritional interventions administered early in life are more effective than similar measures targeting adults^(2)^. Among various interventions, nutrition education has received considerable attention because its impact on improving food choices and enhancing nutrition literacy can be long lasting, even after the intervention ends^(3)^. Proper implementation of nutrition education has been carried out in well-developed countries such as the United Kingdom^(4)^, the United States^(5)^, Germany^(6)^, Poland^(7)^ and Japan^(8)^, demonstrating its effectiveness in improving nutrition-related knowledge, attitudes and dietary habits. Unfortunately, in developing countries such as China, well-designed nutrition education programmes remain scarce^(2)^. The *Report on Chinese Children and Adolescents Nutrition and Health 2014* found that only about 10 % of Chinese children and adolescents had heard of the *Chinese Dietary Guideline* or the *China Food Pagoda* ^(9)^.

Over the past several decades, the Chinese government has attempted to improve the nutritional status of its population through various laws and policies. For example, the *Action Plan for Nutrition Improvement* was issued in 1997 by the State Council of China as part of a national poverty alleviation strategy^(10,11)^. Subsequent policies include the *Management Measures of Nutrition Improvement Work* ^(12)^, the *Outline of China’s Food and Nutrition Development (2014–2020)* ^(13)^, the *Outline of ‘Healthy China 2030’* ^(14)^, the *National Nutrition Plan (2017–2030)* ^(15)^ and, more recently, the *Healthy China Initiative (2019–2030)* ^(16)^. These policies demonstrate strong national commitment and have led to practical guidelines, such as age-specific recipe recommendations and the ‘Exercise + Nutrition’ strategy to maintain healthy weight^(15)^. Despite these efforts, most interventions in China focus on issuing guidelines and regulations for food preparation rather than implementing comprehensive nutrition education programmes^(2)^, To date, only a few programmes, such as the UNICEF-supported ‘Nutrition Campus’ pilot, have been introduced, but their short duration and limited impact on the nutrition literacy of its population remain of concern^(17)^. Consequently, individuals in China often acquire nutrition knowledge from informal sources rather than standardised curricula^(2)^. Among different population groups, university students are particularly important to study because they represent a critical stage between adolescence and adulthood, when dietary habits and nutrition knowledge can influence lifelong health outcomes^(3)^. In China, the absence of standardised nutrition education during earlier schooling means that university students’ nutrition knowledge largely depends on informal sources^(2)^. This study aims to examine university students’ food consumption behaviours and self-perceived nutrition knowledge and to explore the extent to which their confidence in this knowledge aligns with their actual understanding. In other words, a major objective is to investigate the potential ‘illusion of knowing’, a phenomenon in which individuals believe they understand a topic better than they actually do. Understanding this discrepancy can provide insights into the long-term effects of limited nutrition education and inform strategies for future interventions.

## Methods

### Participant recruitment and ethical consideration

This study employed a mixed-methods design combining a self-administered questionnaire with follow-up semi-structured interviews. The purpose of this design was exploratory: to identify participants’ self-perceived nutrition knowledge and to examine, through in-depth interviews, how participants articulated their self-reported knowledge. All participants were recruited in this study by convenience sampling. They were provided with a consent form to ensure that participants involved in this study were totally on a voluntary basis. Before data collection was conducted, participants were informed of their rights to withdraw from the study. The participants were also explained the objective of this study. To secure the privacy and confidentiality of their contributions, a research code was assigned to each of the participants to represent their identity so as to protect their anonymity.

### Quantitative data collection and analysis

A questionnaire was developed to capture participants’ self-declared perceptions of their nutrition knowledge across a range of nutrition-related topics. It was not intended to function as an objective knowledge assessment and therefore did not include scored knowledge questions, predefined correct answers or a total knowledge score. The questionnaire served two purposes: (1) to document participants’ perceived knowledge and (2) to inform the subsequent interviews, where applicable, by identifying topic areas for deeper exploration. For descriptive purposes, the questionnaire was analysed using IBM SPSS Statistics 26·0 for reliability and validity analysis (YES-1, NO-0). Cronbach’s alpha (*α* = 0·808) was calculated to provide information on the internal consistency of the self-perceived knowledge items. These metrics are reported for informational purposes only and are not intended to indicate construct validity as in a traditional knowledge test or attitude scale. Factor analysis was not performed, as the questionnaire was not designed to measure underlying latent constructs.

An electronic version of the questionnaire was distributed via the WeChat platform to 270 recipients who were recruited by convenience sampling. WeChat was used in this study as a platform for distributing the questionnaire because of its high popularity among the Chinese young population across socio-demographic characteristics. A total of 190 questionnaires were gathered. Among them, ninety-four were undergraduate students and ninety-six were postgraduate students. All postgraduate participants were enrolled in Master’s programmes. Data were analysed using IBM SPSS Statistics 26·0. The *χ* ^2^ test was performed to evaluate the intergroup comparisons. A *P* value of < 0·05 was considered to be statistically significant.

### Qualitative data collection

Qualitative data were collected through semi-structured online interviews with a subset of clients who participated in the survey study. Participants were stratified by educational level and then randomly selected for interviews. Around one-third of the participants (*n* 60) were invited, of whom 34 agreed to participate. Interview questions were informed by participants’ questionnaire responses, with particular attention to areas in which participants reported having nutrition knowledge. Participants were invited to explain concepts in their own words, describe how they applied nutrition knowledge in daily life, and discuss sources and reasoning underlying their understanding. The objective of the interviews was to explore the depth, coherence, and accuracy of participants’ expressed knowledge, rather than to test recall of factual information. This qualitative approach allowed participants to demonstrate understanding through explanation and contextualisation.

All interviews were conducted in Mandarin and audio-recorded. Recordings were transcribed verbatim and subsequently translated into English. Interviews were conducted by the first author, who was accredited as a Registered Dietitian in China and a Registered Nutritionist in the United Kingdom at the time of the study. Professional judgment grounded in formal academic training in nutrition was used to assess whether participants demonstrated accurate understanding, misconceptions, or uncertainty in the topic areas they reported knowing. This assessment focused on participants’ reasoning, explanations and use of concepts, rather than on numerical scoring or predefined correct answers.

### Qualitative analysis

Interview transcripts were reviewed to verify participants’ self-declared nutrition knowledge. Transcripts were read and reread to identify statements related to topics each interviewee reported knowing in the questionnaire. Each statement was evaluated to determine whether it demonstrated disciplinary understanding, misconceptions, or uncertainty. Participants were also asked to describe the sources of their knowledge, such as where they learned about the nutrient or its dietary sources. This information provided context to support the evaluation of their self-declared understanding. Statements where participants expressed confidence but provided vague, inconsistent, or inaccurate explanations were noted as discrepancies between perceived and actual knowledge. The analysis was conducted by the first author, with peer checking among the research team to discuss interpretations and ensure consistency in evaluating understanding. Formal inter-coder reliability statistics were not applied, as this part of the study focused on evaluating accuracy and meaning rather than generating themes or quantifying agreement.

### Trustworthiness

The trustworthiness of the study was enhanced by addressing credibility, dependability, and confirmability. Credibility was strengthened through the use of open-ended interview questions, which allowed participants to articulate their self-reported knowledge, and through triangulation of questionnaire and interview data. During interviews, the interviewer paraphrased participants’ responses to ensure accurate interpretation. Dependability was supported through audit trails maintained throughout data collection and analysis, documenting methodological decisions and analytic processes. Confirmability was enhanced through peer checking among the research team, during which interpretations were discussed to reach consensus and minimise individual researcher bias.

## Results

### Demographic characteristics of research participants

As shown in Table 1, a total of 190 students were recruited to complete the questionnaire. Of these, 49·5 % were undergraduates and 50·5 % were postgraduates. Participants’ ages ranged from 18 to 30 years. Among them, 47·4 % were male and 52·6 % were female. To gain deeper insights into the survey findings, interviews were conducted with participants randomly selected from different strata based on educational level. In total, thirty-four participants were interviewed, including ninteen undergraduates (eleven males and eight females) and fifteen postgraduates (eleven males and four females).

**Table 1.** Number of participants and gender distribution across the studied groups

Group	*n*	Gender	*n*
Undergraduate students	94	Male	41
		Female	53
Postgraduate students	96	Male	49
		Female	47

### Awareness of nutritional information on food packages

The nutrition label awareness of individuals with different educational backgrounds is presented in Table 2. When asked whether they read the ingredient list or nutrition facts when purchasing prepackaged foods, 57·4 % of undergraduate students answered ‘yes’, compared to 70·8 % of postgraduate students. This suggests that higher educational attainment may be associated with greater nutritional awareness, leading individuals to pay more attention to food labels when buying prepackaged products. The hypothesis that the likelihood of reading ingredient lists or nutrition facts is linked to educational background is partially supported by interview responses. One postgraduate participant stated, ‘*I took an elective course related to nutritional sciences at my university out of interest and learned how to read labels…this has become my routine practice for food selection since then’.* Another postgraduate participant mentioned, ‘*I read the food label and ingredient list to ensure the food I buy is healthy…this requires some skills and knowledge, but I know that’.* A similar observation was made among undergraduates who had taken courses related to food science and nutrition. One participant said, ‘*Understanding food labels requires some knowledge of nutrition. I learned that as part of my university studies*’.

**Table 2.** Responses to questions on awareness of nutritional information on food packages

Question	Undergraduate students		Postgraduate students		χ^2^	*P*
	Yes (%)	No (%)	Yes (%)	No (%)		
Do you read the ingredient list or nutrition facts list when buying prepackaged foods?	57·4	42·6	70·8	29·2	3·94	0·047
Do you pay attention to descriptions like ‘zero sugar’ or ‘low sugar’ when buying prepackaged foods?	67·0	33·0	69·5	30·5	0·14	0·707
Do you pay attention to descriptions like ‘0 fat’, ‘skimmed’, ‘semi-skimmed’, or ‘whole fat’ when buying prepackaged food?	74·5	25·5	73·2	26·8	0·02	0·898

The lack of prior training in food and nutritional sciences appears to reduce the likelihood that participants read ingredient lists and nutrition facts. This is illustrated by one undergraduate participant who stated, ‘*I am not a science student. I have no idea what the ingredient list and nutrition facts are about. What’s the point of reading them?*’ One interviewee even viewed nutrition claims as primarily marketing messages rather than scientifically grounded information. Promoting nutrition knowledge seems to be an effective way to increase the probability that students read these labels when purchasing prepackaged foods. Although educational level appears positively related to the likelihood of reading ingredient lists and nutrition facts, it does not translate into improved awareness of nutrition claims on food products. This is evident from responses to questions such as, ‘Do you pay attention to descriptions like “zero sugar” or “low sugar” when buying prepackaged foods?’ and ‘Do you pay attention to descriptions like “0 fat”, “skimmed”, “semi-skimmed”, or “whole fat” when buying prepackaged foods?’ Differences between undergraduates and postgraduates regarding concern about added sugar and fat content were not statistically significant.

Furthermore, although around 70 % of participants claimed to pay attention to nutrition claims, the true meaning of these claims was not widely understood. Among the interviewees who reported paying attention to nutrition claims, none could accurately define them. One postgraduate participant said, ‘*A drink labeled “zero sugar” means sugar is absent…if the drink contains sugar, how can it be called “zero sugar”?*’ Similarly, an undergraduate participant stated, ‘*Foods with “zero fat” give no fat and foods with “zero sugar” give no sugar’.* Only one student acknowledged, ‘*I have heard that these claims have definitions and are regulated by law, but I forgot the details…I came across this information online a long time ago’.* It can be observed that participants’ understanding of these claims largely remains at the literal level rather than reflecting their knowledge of regulatory standards. For example, the National Food Safety Standard GB 28050-2011 issued by the Ministry of Health of the People’s Republic of China stipulates that ‘sugar-free’ refers to a sugar content ≤ 0·5 g per 100 g (solid) or 100 mL (liquid), while ‘fat-free’ means fat content ≤ 0·5 g per 100 g (solid) or 100 mL (liquid)^(18)^. This reveals that the illusion of knowing regarding nutrition claims is prevalent among students.

### Variations in nutrition knowledge and understanding

When participants were asked about the roles of carbohydrates in the body, 58·5 % of undergraduates and 58·6 % of postgraduates indicated familiarity (Table 3). Regarding the physiological roles of proteins and fats, 73·4 % of undergraduates and 61·5 % of postgraduates reported knowing the functional role of proteins, while 64·9 % of undergraduates and 57·8 % of postgraduates indicated awareness of the role of fats. The differences between undergraduate and postgraduate students in reported knowledge of these macronutrients were not statistically significant. Qualitative responses from interviews suggest that some students perceive knowledge of macronutrient roles as irrelevant to their current studies or easily forgotten if not regularly applied. For example, one undergraduate participant stated, ‘*I have not dealt with any science-related content since graduating from secondary school…stuff like this is not needed for my current study’.* Similarly, a postgraduate participant commented, ‘*Knowledge of macronutrients is not relevant to my major. If one does not use something regularly, it is common to forget’.* These responses indicate that practical relevance, recency of learning, continued exposure and personal motivation may influence nutrition knowledge among students. Regarding dietary fibre, 77·7 % of undergraduates and 71·8 % of postgraduates reported understanding its role in the body. Compared with carbohydrates, proteins and fats, a slightly higher proportion of students appeared confident about the role of fibre in the diet. This may be partly due to the prominent emphasis on dietary fibre in public health messages and mass media, as well as its relatively simple physiological functions.

**Table 3.** Responses to questions on nutrition knowledge and understanding

Question	Undergraduate students		Postgraduate students		χ^2^	*P*
	Yes (%)	No (%)	Yes (%)	No (%)		
Do you know the food source of carbohydrates?	75·5	25·5	71·6	28·4	0·28	0·599
Do you know the physiological functions of carbohydrates?	58·5	41·5	58·6	41·4	0·01	0·929
Do you know the food source of protein?	78·7	21·3	73·2	26·8	0·89	0·345
Do you know the physiological function of protein?	73·4	26·6	61·5	38·5	2·84	0·092
Do you know the food source of fat?	86·2	13·8	74·8	25·2	3·79	0·052
Do you know the physiological function of fat?	64·9	35·1	57·8	42·2	1·04	0·308
Do you know the food source of dietary fibre?	81·9	18·1	78·5	21·5	0·36	0·549
Do you know the physiological function of dietary fibre?	77·7	22·3	71·8	28·2	0·97	0·325

### Translation of nutrition knowledge into practice

To understand participants’ daily food selection and preparation habits, they were asked whether they prioritise healthy snacks or drinks when purchasing such items (Table 4). Among them, 73·4 % of undergraduates and 69·9 % of postgraduates indicated a preference for healthy snacks and drinks. The difference between the two groups was not statistically significant. When asked whether they deliberately reduce salt when cooking, 44·7 % of undergraduates and 59·9 % of postgraduates responded affirmatively. The proportion of postgraduates answering ‘Yes’ was statistically higher than that of undergraduates, suggesting that postgraduates may be more attentive to salt reduction during cooking. One postgraduate student stated during the interview, ‘*Cutting down salt intake can prevent hypertension. This is repeatedly mentioned in the mass media’.* Another participant added, ‘*Taking too much salt is not good for health’.* However, when the interviewees were asked about the recommended daily intake of salt (6 g per day according to the *Dietary Guidelines for Chinese Residents* prepared by the Chinese Nutrition Society and the Institute of Nutrition and Food Hygiene affiliated with the Chinese Academy of Preventive Medicine)^(19,20)^, none could provide the correct answer.

**Table 4.** Responses to questions on food consumption behaviour

Question	Undergraduate students		Postgraduate students		χ^2^	*P*
	Yes (%)	No (%)	Yes (%)	No (%)		
Do you deliberately cut down on salt when cooking?	44·7	55·3	59·9	40·1	4·21	0·040
Do you deliberately cut down on oil when cooking?	72·3	27·7	84·1	15·9	4·06	0·044
Do you prioritise healthy snacks or drinks when buying snacks or drinks?	73·4	26·6	69·9	30·1	0·22	0·636
Do you deliberately control how full you feel at every meal?	46·8	53·2	53·5	46·5	0·79	0·373
Do you deliberately control your eating time?	41·5	58·5	49·0	51·0	0·93	0·335

A similar pattern was observed for oil intake. While 72·3% of undergraduates and 84·1 % of postgraduates indicated that they deliberately control oil content during food preparation, none of the interviewed participants who reported controlling oil intake could state the recommended daily intake (25–30 g) as specified in the *Dietary Guidelines for Chinese Residents* ^(20)^. Participants were also asked whether they deliberately control satiety at each meal and regulate their eating time. Both undergraduates and postgraduates reported paying attention to these factors. Among those interviewed who indicated controlling eating time and satiety, approximately 58·8 % mentioned having dieted before. More than 80 % of interviewed female participants regarded dieting as a strategy to remain slim, associating slimness with beauty. One participant stated, ‘*Controlling diet can control food intake. Eating too much will lead to obesity…being slim looks good, and everybody likes to look good’.* Another female participant added, ‘*I have been on a diet several times. Keeping fit is a lifelong career for women, and we need to control the amount of food we eat every day’.* In contrast, male participants tended to emphasise the health benefits of proper diet rather than linking dieting solely to slimness. This was reflected in one participant’s comment: ‘*Eating too much will cause diabetes and many diseases. I do not want to have these diseases when I get old’.*

## Discussion

Food selection is a key domain related to nutrition literacy and is a complex process influenced not only by cognitive factors but also by biological and environmental factors that shape preferences, habits and responses to rewarding stimuli^(21)^. To support healthier food choices, information about food products is mandatorily disclosed on prepackaged foods through labels that include text, graphics and nutrient descriptions. These labels serve as a public health policy tool, providing information about ingredients and nutrients such as energy, protein, fat, sugar and salt that may otherwise be unknown to consumers^(22)^. Previous studies have shown that consumers generally perceive food labels as a credible source of information and report using them to guide food selection^(23)^. Use of food labels has also been associated with healthier dietary patterns, including reduced energy intake when calorie information is provided at the point of purchase^(24,25)^. In this context, attention to nutrition labels among university students may serve as an indirect indicator of nutrition literacy as well as the reach of nutrition promotion efforts.

Findings from the present study, however, indicate that habitual attention to nutrition labels does not necessarily reflect adequate understanding of the information presented. Although many participants reported reading nutrition claims during food purchases, interviews revealed that they often lacked a clear understanding of the regulatory definitions of these claims. This discrepancy highlights a potential illusion of knowing, whereby individuals believe they understand nutritional information better than they actually do. It may lead people to rely on inaccurate interpretations of nutritional information and may prevent them from making choices that align with their health needs. As revealed in this study, educational attainment appeared to influence engagement with nutrition labels, with students at higher educational levels reporting greater attention to ingredient lists and nutrition claims. However, this increased attention did not consistently translate into accurate comprehension. This finding suggests that current nutrition labelling practices may require a foundational level of nutrition knowledge to be fully understood, a level that may not be uniformly present among students. Improving the accessibility and interpretability of nutrition labels, together with strengthening nutrition education, may therefore be necessary to enhance the effectiveness of nutrition labelling across diverse populations.

Misconceptions surrounding nutrition claims observed in this study may be partly linked to the absence of standardised nutrition education within formal schooling in China^(2)^. National data indicate limited awareness of authoritative dietary guidance among individuals in China, with only a small proportion having heard of the *Chinese Dietary Guidelines* or the *China Food Pagoda* ^(9)^. Although students in this study demonstrated general nutrition-related awareness, the knowledge expressed during interviews was often fragmented, reinforcing susceptibility to the illusion of knowing in the absence of systematic training. Qualitative data further illustrated skepticism toward nutrition claims, with one interviewee describing such claims as primarily advertising tactics. While nutrition claims are regulated by law and differ from purely promotional statements, misunderstanding their purpose and meaning may discourage their appropriate use during food selection. These findings underscore the need for nutrition education initiatives that clarify the role, regulation and interpretation of nutrition claims.

## Concluding remarks

Nutrition literacy plays an important role in shaping food selection behaviours and long-term health outcomes^(26)^. Establishing sound nutrition knowledge and appropriate food consumption practices among students is therefore important for enhancing nutrition literacy and may contribute to reducing the risk of nutrition-related chronic diseases such as diabetes and CVD later in life^(26)^. Nutritional interventions targeting the general public during the student years have long been recognised as an effective strategy for health promotion^(27–29)^. In this study, China served as a case study to explore university students’ self-perceived nutrition knowledge and their interpretation of nutritional information in a context where formal nutrition education before university is limited. Our findings suggest that, in addition to promoting students’ attention to nutritional information, fostering their deeper understanding of nutrition concepts is pivotal. Apart from establishing standardised and systematic nutrition curricula for student populations, adequate allocation of educational resources and training of qualified nutrition educators are essential for effective nutrition education^(2)^.

Despite careful study design, several limitations of this study should be noted. The use of a convenience sampling method may introduce response bias and limit the generalisability of findings beyond the study sample. Interviews were conducted to verify self-declared nutrition knowledge; however, this assessment inevitably involves subjective judgement, as interpretations were dependent on the interviewer. Furthermore, owing to the breadth of nutritional science and the limited scope of the questionnaire, only selected major nutrition topics were examined. Other relevant aspects of students’ dietary knowledge or practices may not have been captured. Future research employing broader topic coverage and more diverse populations across age groups, socio-economic backgrounds, cultures and countries would help provide a more comprehensive understanding of food consumption behaviour and self-perceived nutrition knowledge among individuals with limited formal nutrition education during pre-university schooling.

## Supporting information

10.1017/S1368980026102468.sm001 Lai et al. supplementary material Lai et al. supplementary material

## Supplementary material

For supplementary material accompanying this paper visit https://doi.org/10.1017/S1368980026102468
