# Supplementary material for: Food consumption behaviour and self-perceived nutrition knowledge: a case study of students with limited formal nutrition education in pre-university schooling
Source: Public Health Nutr. 2026 Apr 10;29(1):e98. doi: 10.1017/S1368980026102468 (PMC13430539; doi:10.1017/S1368980026102468)
Supplement: Lai et al. supplementary material [file S1368980026102468sup001.docx]

**Questionnaire on Dietary Consumption Behavior and Self-Declared Nutritional Cognition**

| Gender: | 🞏 Male | 🞏 Female |
| --- | --- | --- |
| Age: | ____________________ | |
| Ethnicity: |  | |
| Educational level: | 🞏 Undergraduate | 🞏 Postgraduate |
| Height: | ____________________ cm | |
| Weight: | ____________________ kg | |

| 1. Do you read the ingredient list or nutrition facts list when buying prepackaged foods? | 🞏 Yes | 🞏 No |
| --- | --- | --- |
| 1. Do you pay attention to descriptions like ‘zero sugar’ or ‘low sugar’ when buying prepackaged foods? | 🞏 Yes | 🞏 No |
| 1. Do you pay attention to descriptions like ‘0 fat’, ‘skimmed’, ‘semi-skimmed’, or ‘whole fat’ when buying prepackaged food? | 🞏 Yes | 🞏 No |
| 1. Do you know the food source of carbohydrates? | 🞏 Yes | 🞏 No |
| 1. Do you know the nutritional physiological functions of carbohydrates? | 🞏 Yes | 🞏 No |
| 1. Do you know the food source of protein? | 🞏 Yes | 🞏 No |
| 1. Do you know the physiological function of protein? | 🞏 Yes | 🞏 No |
| 1. Do you know the food source of fat? | 🞏 Yes | 🞏 No |
| 1. Do you know the physiological function of fat? | 🞏 Yes | 🞏 No |
| 1. Do you know the food source of dietary fiber? | 🞏 Yes | 🞏 No |
| 1. Do you know the physiological function of dietary fiber? | 🞏 Yes | 🞏 No |
| 1. Do you deliberately cut down on salt when cooking? | 🞏 Yes | 🞏 No |
| 1. Do you deliberately cut down on oil when cooking? | 🞏 Yes | 🞏 No |
| 1. Do you prioritize healthy snacks or drinks when buying snacks or drinks? | 🞏 Yes | 🞏 No |
| 1. Do you deliberately control how full you feel at every meal? | 🞏 Yes | 🞏 No |
| 1. Do you deliberately control your eating time? | 🞏 Yes | 🞏 No |
